# Arsenic Modulates Posttranslational S-Nitrosylation and Translational Proteome in Keratinocytes

**DOI:** 10.1155/2014/360153

**Published:** 2014-07-08

**Authors:** Bin Huang, Kuo-Hao Chiang, Hsin-Su Yu, Ying-Lun Chen, Huey-Ling You, Wei-Ting Liao

**Affiliations:** ^1^Department of Biomedical Science and Environmental Biology, College of Life Science, Kaohsiung Medical University, Kaohsiung 80708, Taiwan; ^2^Department of Biological Sciences, National Sun Yat-sen University, Kaohsiung 80424, Taiwan; ^3^Department of Biotechnology, College of Life Science, No. 100, Shihchuan 1st Rd., Sanming District, Kaohsiung Medical University, Kaohsiung 80708, Taiwan; ^4^Department of Dermatology, Kaohsiung Medical University Hospital and Kaohsiung Medical University College of Medicine, Kaohsiung 80708, Taiwan; ^5^Department of Anesthesiology, Mackay Memorial Hospital, Taipei 10449, Taiwan; ^6^Departments of Laboratory Medicine, Chang Gung Memorial Hospital-Kaohsiung Medical Center, Kaohsiung 83301, Taiwan

## Abstract

Arsenic is a class I human carcinogen (such as inducing skin cancer) by its prominent chemical interaction with protein thio (-SH) group. Therefore, arsenic may compromise protein S-nitrosylation by competing the -SH binding activity. In the present study, we aimed to understand the influence of arsenic on protein S-nitrosylation and the following proteomic changes. By using primary human skin keratinocyte, we found that arsenic treatment decreased the level of protein S-nitrosylation. This was coincident to the decent expressions of endothelial nitric oxide synthase (eNOS) and inducible nitric oxide synthase (iNOS). By using LC-MS/MS, around twenty S-nitrosoproteins were detected in the biotin-switched eluent. With the interest that arsenic not only regulates posttranslational S-nitrosylation but also separately affects protein's translation expression, we performed two-dimensional gel electrophoresis and found that 8 proteins were significantly decreased during arsenic treatment. Whether these decreased proteins are the consequence of protein S-nitrosylation will be further investigated. Taken together, these results provide a finding that arsenic can deplete the binding activity of NO and therefore reduce protein S-nitrosylation.

## 1. Introduction

Protein S-nitrosylation, the covalent attachment of nitric oxide (NO) to protein thio (-SH) group, is an important posttranslational modification that affects a wide variety of proteins for cellular signaling in normal physiology and a broad spectrum of human diseases [1, 2]. S-nitrosylation signaling controls a number of cellular processes, such as protein-protein interactions [3], nuclear transcriptions [4], and membrane-associated proteins activation [5, 6]. Pathophysiology is correlated with hypo- or hyper-S-nitrosylation of specific protein targets rather than a general cellular insult due to not only the loss of or enhanced nitric oxide synthase activity but also the denitrosylation by a major denitrosylase, S-nitrosoglutathione reductase (GSNOR) [1]. Abnormal protein S-nitrosylation causes many diseases such as cardiovascular, musculoskeletal, and neurological dysfunction [7]. Furthermore, autophagy, a vacuolar degradation for long-lived and aggregate-prone proteins, plays an important role in neurodegeneration. Inhibition of autophagy by S-nitrosylation results in stress-mediated protein aggregation in neurodegenerative diseases [8, 9].

S-nitrosylation is also associated with cancer [10]. Potential mechanisms of S-nitrosylation in carcinogenesis are focused on apoptosis and DNA repair [11]. Survival of tumor cell could be induced by inactivation of proapoptotic signaling or activation of antiapoptotic pathways [12]. Inhibition of caspase protease by protein nitrosylation promotes extended survival of malignant cells [13]. For example, nitrosylation of caspase 9 inhibits the mitochondrial pathway of apoptosis in cholangiocarcinoma cell line [14]. Additionally, p53 induces apoptotic cell death and causes cell cycle arrest in response to various stresses [15]. S-nitrosylation of p53 suppresses p53-mediated apoptosis in colon carcinogenesis [16]. Bcl-2, a major anti-apoptotic regulatory protein, was regulated by S-nitrosylation in various carcinoma tissues [11, 17, 18]. S-nitrosylation is shown to modulate the activity, stability, and cellular localization of key DNA repair proteins, including O(6)-alkylguanine-DNA-alkyltransferase (AGT), 8-oxoguanine glycosylase (Ogg1), apurinic-apyrimidinic endonuclease 1 (APE1), and DNA-dependent protein kinase catalytic subunit (DNA-PKcs) [19]. Inactivation of AGT by S-nitrosylation is found in hepatocarcinogenesis [20]. Moreover, S-nitrosylated APE1 export from the nucleus to the cytoplasm is described in colon adenomas, breast cancer, and hepatocellular carcinomas [21]. Also, S-nitrosylated DNA-PKcs shows increased transcriptional expression and activity in HEK-293 [22].

It is well known that arsenic, a human carcinogen, showed its chemical carcinogenesis activity by interaction with protein -SH groups. By its -SH binding activity, therefore, arsenic may compromise protein S-nitrosylations in cells. In addition, it is reported that arsenic has a significant effect on NO production in the endothelium [23]. Arsenic remains one of the most concerned environmental toxicants, which has been classified as a group I human carcinogen by the International Agency for Research on Cancer (IARC). Epidemiological studies demonstrated that long-term exposure to inorganic arsenic through either ingestion or inhalation is associated with an increased risk of malignant cancers in the urinary bladder, lung, and especially skin, since arsenic tends to concentrate in keratinocytes [24]. However, the role of S-nitrosylation induced by arsenic in skin carcinogenesis remains unclear. In the present study, we hypothesize that arsenic can alter protein S-nitrosylation and NO production in skin keratinocyte. Our contribution is identifying interactions between arsenic and S-nitrosylation axis in keratinocytes that can provide the novel molecular and pharmacological strategies for potential clinical applications.

## 2. Materials and Methods

### 2.1. Human Keratinocyte Culture

Freshly obtained prepuce specimens were used to cultivate the primary human cultured keratinocytes. Briefly, normal human prepuce specimens were washed with PBS, then cut into small pieces, and incubated in medium containing 0.25% trypsin overnight at 4°C. The epidermal sheet was lifted from the dermis using a fine forceps. The epidermal cells were pelleted by centrifugation (500 ×g, 10 min) and dispersed into individual cells by repeated aspiration with a pipette. Isolated keratinocytes must be cultured in commercialized keratinocyte serum free medium (Invitrogen, Carlsbad, USA) at 37°C in a humidified incubator with 5% CO_2_ atmosphere with or without sodium arsenite (Sigma, St. Louis, USA) treatment (10 *μ*M). This primary keratinocyte culture protocol using human skin samples has been approved by Institutional Review Board of Kaohsiung Medical University Hospital (KMUH-IRB-960119).

### 2.2. Cell Lysis and Proteins Extraction

Keratinocytes after treatment were washed with cord buffer [NaCl (0.14 M), KCl (4 mM), glucose (11 mM), and HEPES (10 mM, pH 7.4)] and then lysed with 100 *μ*L of lysis buffer [Hepes (250 mM, pH 7.7), EDTA (1 mM), neocuproine (0.1 mM), and CHAPS (0.4%, w/v)]. After centrifugation, protein supernatant is collected and protein concentrations are determined with BCA assay reagent (Thermo Fisher Scientific Inc, Rockford, IL, USA).

### 2.3. Biotin Switch Method for Purifying S-Nitrosoproteins

The biotin switch method was used according to previous study [22, 25]. Cells were washed with 1 × cord buffer (10 mM HEPES, pH 7.4, 0.14 M NaCl, 4 mM KCl, and 11 mM glucose). Protein lysates were obtained using ultrasound and lysis buffer (250 mM HEPES, pH 7.7, 1 mM EDTA, 0.1 mM neocuproine, and 0.4% (w/v) CHAPS). The free thiols were methylated with blocking buffer (225 mM HEPES, pH 7.7, 0.9 mM EDTA, 0.09 mM neocuproine, 2.5% (w/v) SDS, and 20 mM MMTS) at the ratio of 0.8 mg/1 mL and were incubated at 50°C for 20 min with agitation. To remove residual MMTS, the MMTS-treated lysate was precipitated with cold acetone and the resulting pellet was resuspended in HENS buffer (250 mM HEPES, pH 7.7, 1 mM EDTA, 0.1 mM neocuproine, and 1% (w/v) SDS). This was followed by addition of one-third of the HENS suspension's final volume of 4 mM N-[6-(biotinamido) hexyl]-3′-(2′-pyridyldithio) propionamide (biotin-HPDP/DMF) mixed with 1 mM ascorbate. The protein lysate/biotin-HPDP mixture was incubated at room temperature for 1 hour to allow biotinylation to occur. These mixtures were precipitated with cold acetone to remove excess biotin-HPDP and then resuspended in HENS buffer. The biotinylated proteins (i.e., the former S-nitrosoproteins) were recovered using neutravidin-agarose beads (15 *μ*L/per mg of initiated protein input) in neutralization buffer (20 mM HEPES, pH 7.7, 100 mM NaCl, 1 mM EDTA, and 0.5% (v/v) Triton X-100). The agarose beads were rinsed with washing buffer (20 mM HEPES, pH 7.7, 600 mM NaCl, 1mMEDTA, and 0.5% (v/v) Triton X-100). The biotinylated proteins were eluted by elution buffer (20 mM HEPES, pH 7.7, 100 mM NaCl, 1 mM EDTA, and 100 mM 2-ME). We added 100 femtomole of BSA in the eluent as a standard in the subsequent MS/MS analysis. The salt and detergent in eluents were removed by SpinOUT and DetergentOUT resins (Geno Technology Inc., MO, USA).

### 2.4. Mass Spectrometric Assay

Around 100 ng eluents were reduced, alkylated, and trypsin digested according to the user's guideline (In-Gel Tryptic Digestion Kit, Thermo Fisher Scientific, IL, USA). The peptide lysates were further desalting with Proteomics C18 Column (Mass solution Ltd., Taipei, Taiwan) and then subjected to mass analysis by nLC/Q-TOF tandem mass spectrometry (Micromass, Manchester, UK). MS data were searched for against the NCBI database using an in-house MASCOT search program (Matrix Science, London, UK). Search parameters were set as mass values: monoisotopic, protein mass: unrestricted, peptide mass tolerance: ±0.4 Da, fragment mass tolerance: ±0.4 Da, max missed cleavages: 1, and the instrument type: ESI-QUAD-TOF.

### 2.5. Western Blot Analysis

Forty micrograms of cell lysates with various treatments was mixed with equal volume of sample buffer [Tris-HCl (62.5 mM, pH6.8), SDS (3%, w/v), 2-mercaptoethanol (5%, v/v), and glycerol (10%, v/v)] and then separated by SDS-PAGE. The gel was transferred to PVDF membranes (Millipore, MA, USA) and immunoblotted with antibodies: eNOS (1 : 3000, Cell Signaling Tech. MA, USA), iNOS (1 : 3000, Cell Signaling Tech. MA, USA). The membranes were visualized with the SuperSignal West Femto reagent (Thermo Fisher Scientific, IL, USA) on X-ray films. The images on X-ray films were scanned using a digital scanner (Microtek International Inc.) and the density was calculated by the Progenesis Samespots v2.0 software (NonLinear Dynamics, Newcastle, UK).

### 2.6. Two-Dimensional Gel Electrophoresis

Extracted protein (1 mg) was precipitated with 3 volumes of cold acetone at −20°C for at least 20 min. After centrifugation, the protein pellets were air-dried for 5 min, dissolved in sample buffer [9 M urea, 2% (w/v) CHAPS, 60 mM DTT, and 2% (v/v, pH 4–7) IPG buffer (GE Healthcare BioSci., NJ, USA)], and incubated for at least 30 min to denature proteins completely. The protein solution was mixed with rehydration solution [8 M urea, 2% (w/v) CHAPS, and 0.5% (v/v, pH 4–7) IPG buffer] to reach a final volume of 340 *μ*L and then soaked into an 18 cm DryStrip (pH 4–7, GE Healthcare BioSci.) for up to 12 h on Ettan IPGphor system (GE Healthcare BioSci.). Isoelectric focusing (IEF) was performed with the accumulated voltage set to 32 kVh. After IEF analysis, stripped gels were equilibrated with trisbuffer [50 mM Tris-HCl, pH 8.8, containing 2% (w/v) SDS, 6 M urea, 30% (v/v) glycerol, and 60 mM DL-dithiothreitol (DTT)] for 20 min. The stripped gels were then alkylated in the same buffer containing 135 mM iodoacetic acid for additional 20 min. The equilibrated IEF strip was laid on the top of a vertical SDS-PAGE system to perform the 2-DE.

### 2.7. Image Analysis

The gels were stained with VisPRO dye (Visual Protein Biotech., Taipei, Taiwan) and scanned with a digital scanner (Microtek International Inc.). The translational levels were calculated by ImageMaster software (GE Healthcare BioSci.). The gel slices excised from the silver-stained gel were digested with trypsin for 4 hours at 37°C (In-Gel Tryptic Digestion Kit, Thermo Fisher Scientific Inc.) and then subjected to mass spectrometric analysis.

## 3. Results and Discussion

### 3.1. Arsenic Reduces Protein S-Nitrosylation

**Figure 1:**
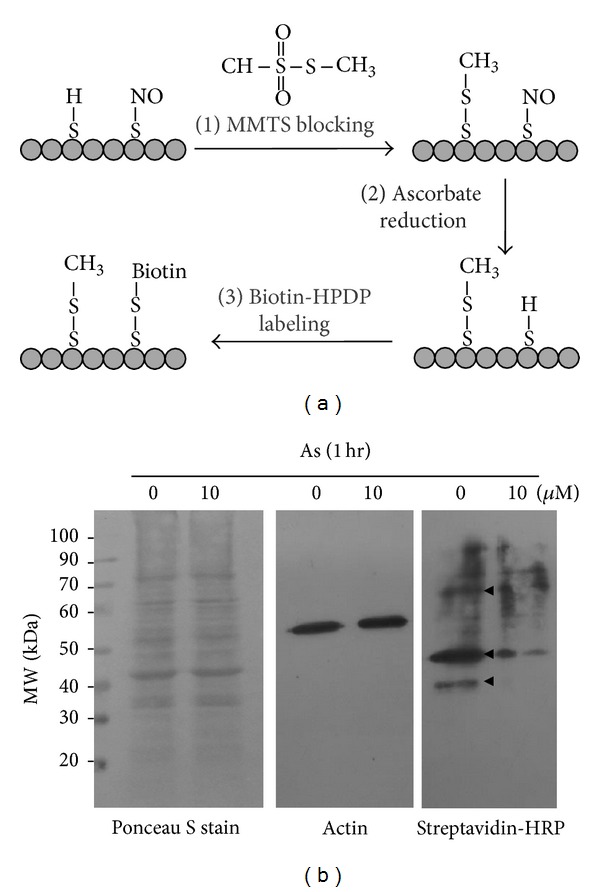
The arsenic decreases protein S-nitrosylation in primary keratinocytes. (a) The scheme of biotin switch showed that the NO on the cysteine residue was replaced by biotin. (b) After biotin switch, 40 *μ*g of biotinylated lysates treated with 10 *μ*M of arsenic for 1 h was separated by SDS-PAGE. The blotted membrane was prestained by Ponceau S and western blotted with actin (1 : 3000). Both two were applied as loading control. Streptavidin-HRP (1 : 3000) was applied to detect biotinylated proteins. The decreased S-nitrosylated proteins after arsenic treatment were indicated as triangle.

Biotin switch is now the most popular methodology for identifying S-nitrosoproteins in various tissues [22]. In the present study, the procedure of biotin switch was simply indicated (Figure 1(a)). By treatment with 10 *μ*M of arsenic for 1 hour, at least three groups of protein S-nitrosylation were significantly decreased in keratinocytes (Figure 1(b)). These biotinylated proteins, that is, NO-bound S-nitrosylated proteins, were purified from streptavidin conjugated agarose and then analyzed by LC-MS/MS. Totally, 20 and 13 S-nitrosoproteins were separately identified in the control and arsenic treatments. This was coincident to the decreased NO production and the reduced S-nitrosylated proteins.

### 3.2. Arsenic Decreases the Expressions of eNOS and iNOS

**Figure 2:**
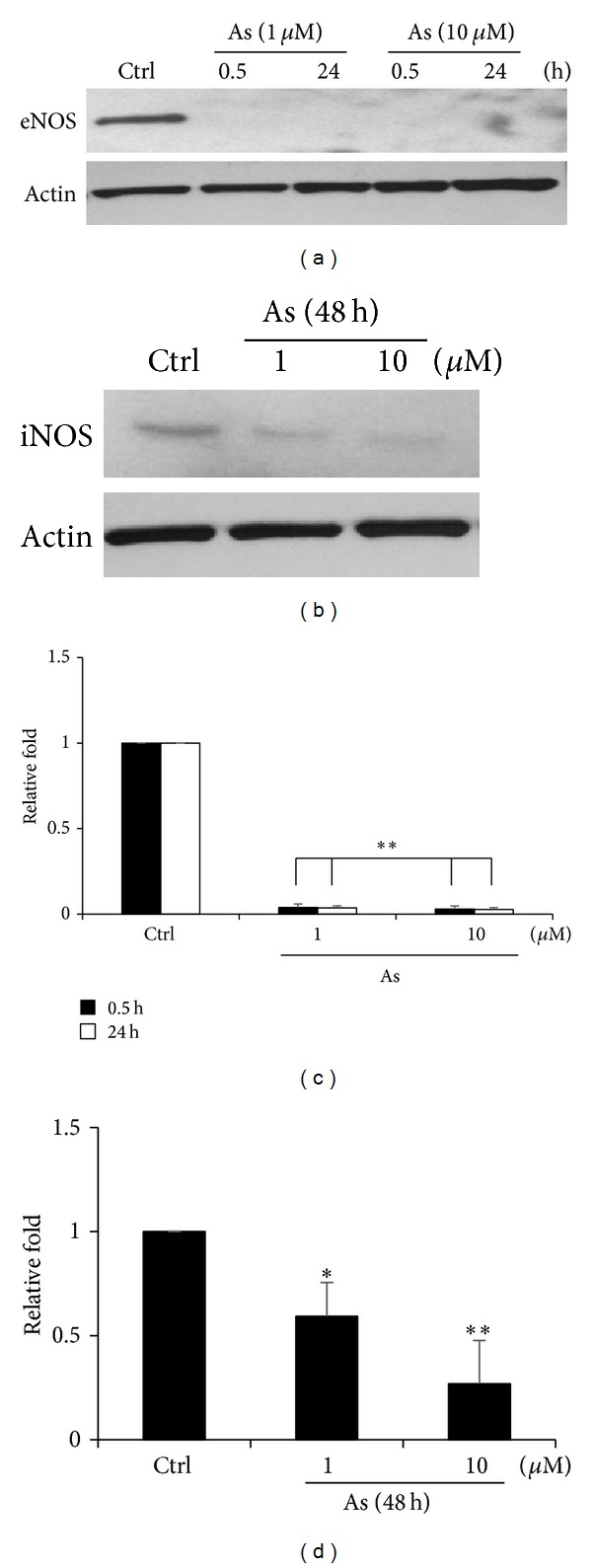
Arsenic attenuates the expressions of eNOS and iNOS. (a) Cells treated with 1 or 10 *μ*M of arsenic for 0.5, 24 were used to monitor eNOS variations. (b) For investigating iNOS level, the cells were incubated with same concentration of arsenic for 48 hours. (c, d) The expression levels of eNOS and iNOS were statistically calculated from three repeats. Relative folds of protein levels shown as means ± S.E. compared to control. Statistical significance (*P < 0.05, **P < 0.01) analyzed using Fisher's LSD.

It has been reported that NO in keratinocytes was produced through the activation of eNOS and iNOS [23]. Therefore, in the present study, we examined the expressions of both enzymes in the existence of arsenic. As shown in Figures 2(a) and 2(b), the significant decreases of their expressions were observed.

### 3.3. Arsenic Modulates Translational Proteome in Keratinocytes

**Figure 3:**
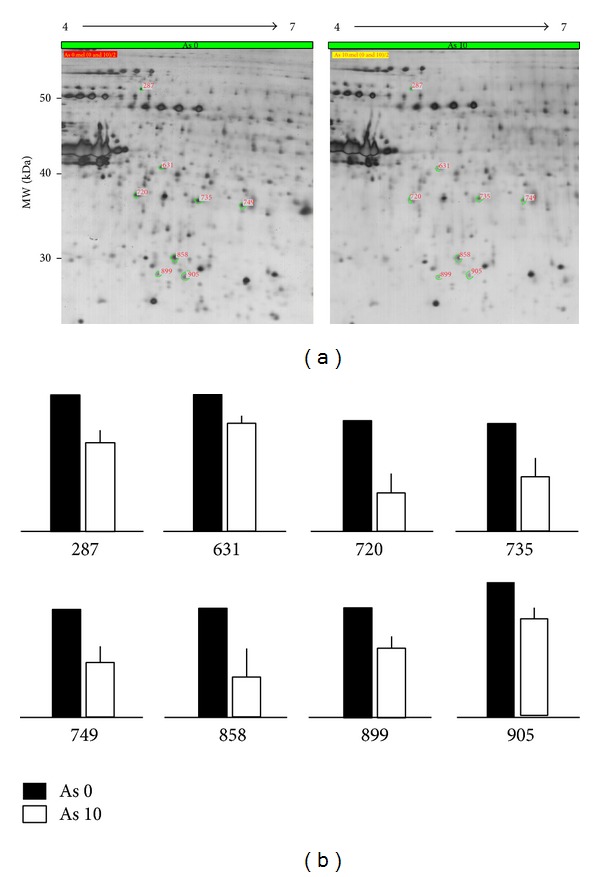
Translational proteome regulated by arsenic. The cell lysates (1 mg) treated with 10 *μ*M of arsenic for 1 h were separated by 2-DE (pH4–7). By using ImageMaster software, the proteins with decreased expression (<0.7 fold) were indicated. (b) The relative expression levels of these decreased proteins were statistically calculated from three repeats.

In addition to elucidating those posttranslational S-nitrosylated proteins, we further investigated the proteins changed in their expression levels. By using 2-DE, 8 proteins showed a dramatic decrease in the expression level (Figures 3(a) and 3(b)). These identified proteins are annotated function in (Table 1). More specifically, HNRNPK is a pre-mRNA-binding protein, which has been identified to be involved in arsenic-induced apoptosis in acute myeloid leukemia cells [26]. PSMD13 is proteasome protein regulating the degradation of ubiquitinated proteins, which has been identified in fetal fibroblasts from glutathione deficient mouse using arsenic-induced apoptosis model by cDNA microarray [27]. RPLP0 is a ribosomal protein, in general conditions, constantly expressed in cells (house-keeping gene). The functional interactions between arsenic and RPLP0 required further investigations. CTSD is a protease active in intracellular protein breakdown. Increased CTSD has been identified in arsenic-treated human lymphoblastoid cell lines correlated with autophagy [28]. However, here, we found arsenic treatment decreased CTSD in human keratinocytes. NDUFS3 is a subunit of the mitochondrial membrane respiratory chain NADH dehydrogenase (Complex I). Decreased NDUFS3 has been identified in hepatoma cell line [29]. Indeed, our previous study has identified the dose response between arsenic and NDUFS3 in human keratinocytes. Low concentrations (0.1–1.0 *μ*M) of arsenic increased NDUFS3 associated with keratinocyte proliferation. High concentration (5.0 *μ*M) of arsenic decreased NDUFS3 correlated with cell death [30]. This decreased NDUFS3 was represented in our current data. ECHS1 is a catalytic enzyme in mitochondrial fatty acid betaoxidation pathway; however, direct evidence between ECHS1 and arsenic is not yet clarified. To summarize, from our proteomic data, altered apoptosis and mitochondrial metabolic functions were the dominant effects of arsenic on human keratinocytes.

## 4. Conclusion

In the current study, we concluded that arsenic can compete with nitric oxide in binding cysteine residues so that the protein S-nitrosylation is inhibited. Whether the decreased S-nitrosylation is correlated to arsenic-induced pathology attracts a great attention in the further study. In addition, the homeostasis of cellular apoptosis and mitochondrial dysfunctions is worthy of further study.

## Figures and Tables

**Table 1:** Identification of arsenic-modulated proteins with nLC-MS/MS.

Spot number	Protein name^a^	Accession number^b^	MW (kDa)/pI Thero.^c^	MW (kDa)/pI Exp.^d^	Sequence coverage (%)	MOWSE score	Peptides Matched
287	Transformation upregulated nuclear protein	460789	51.0/5.1	54.5/4.9	12	268	3

631	26S proteasome non-ATPase regulatory subunit 13 isoform 1	157502193	42.9/5.5	44.3/5.2	19	380	8

720	60S acidic ribosomal protein P0	4506667	34.2/5.7	39.2/5.0	18	326	7

735	60S acidic ribosomal protein P0	4506667	34.2/5.7	39.2/6.2	29	422	7

749	60S acidic ribosomal protein P0	4506667	34.2/5.7	38.9/6.5	36	636	9

858	Cathepsin D preproprotein	4503143	44.5/6.1	30.4/5.3	25	422	9

899	NADH-Ubiquinone reductase	4758788	30.2/6.9	26.4/5.8	40	575	8

905	Enoyl-CoA hydratase	1922287	31.3/8.3	26.4/6.1	16	180	3

^a^Function of the protein obtained via the MASCOT software (http://www.matrixscience.com) search program by querying the NCBI database. The parameters were set at peptide mass tolerance ±0.4 Da and allowed missed cleavage 1.

^b^Accession number from NCSI database.

^c^Theoretic protein molecular weight and pI annotated in NCBI database.

^d^Experimental protein molecular weight and pI calculated from 2-DE gel.
